# Comparative study of three plant-derived extracts as new management strategies against *Spodoptera littoralis* (Boisd.) (Lepidoptera: Noctuidae)

**DOI:** 10.1038/s41598-023-30588-x

**Published:** 2023-03-02

**Authors:** Hanaa S. Hussein, Mohamed Z. M. Salem, Ahmed M. Soliman, Sahar E. Eldesouky

**Affiliations:** 1 Applied Entomology and Zoology Department, Faculty of Agriculture (EL-Shatby), Alexandria University, Alexandria, 21545 Egypt; 2 Forestry and Wood Technology Department, Faculty of Agriculture (EL-Shatby), Alexandria University, Alexandria, 21545 Egypt; 3 Cotton Pesticides Evaluation Department, Plant Protection Research Institute, Agricultural Research Center, El-Sabhia, Alexandria, Egypt

**Keywords:** Biochemistry, Biological techniques, Zoology

## Abstract

Finding innovative eco-friendly agents for pest control may be aided by investigating the plant-derived extracts’ properties on economic pests. Therefore, the insecticidal, behavioral, biological and biochemical effects of *Magnolia grandiflora* (Magnoliaceae) leaf water and methanol extracts, *Schinus terebinthifolius* (Anacardiaceae) wood methanol extract, and *Salix babylonica* (Salicaceae) leaf methanol extract in comparison with a reference insecticide novaluron against *S. littoralis* were evaluated. The extracts were analyzed by High-Performance Liquid Chromatography (HPLC). The most abundant phenolic compounds were 4-hydroxybenzoic acid (7.16 mg/mL) and ferulic acid (6.34 mg/mL) in *M. grandiflora* leaf water extract; catechol (13.05 mg/mL), ferulic acid (11.87 mg/mL), and chlorogenic acid (10.33 mg/mL) in *M. grandiflora* leaf methanol extract; ferulic acid (14.81 mg/mL), caffeic acid (5.61 mg/mL), and gallic acid (5.07 mg/mL) In the *S. terebinthifolius* extract; cinnamic acid (11.36 mg/mL), and protocatechuic acid (10.33 mg/mL) In the methanol extract from *S. babylonica* extract. *S. terebinthifolius* extract had a highly toxic effect against second larvae after 96 h and eggs with LC_50_ values of 0.89 and 0.94 mg/L, respectively. Despite *M. grandiflora* extracts didn’t show any toxicity against *S. littoralis* stages, they had an attractant effect on fourth- and second larvae, with feeding deterrence values of − 2.7% and − 6.7%, respectively, at 10 mg/L. *S. terebinthifolius* extract significantly reduced the percentage of pupation, adult emergence, hatchability, and fecundity, with values of 60.2%, 56.7%, 35.3%, and 105.4 eggs/female, respectively. Novaluron and *S. terebinthifolius* extract drastically inhibited the activities of α-amylase and total proteases to 1.16 and 0.52, and 1.47 and 0.65 ΔOD/mg protein/min, respectively. In the semi-field experiment, the residual toxicity of tested extracts on *S. littoralis* gradually decreased over time compared to novaluron. These findings indicate that extract from *S. terebinthifolius* is a promising insecticidal agent against *S. littoralis*.

## Introduction

The cotton leafworm, *Spodoptera littoralis* (Boisd) (Lepidoptera: Noctuidae), is the major destructive pest of several agricultural crops including cotton, eggplant, tomato, and some ornamental products in Africa, Mediterranean Europe and Middle Eastern countries. More than 100 host attacked by this pest species, which causes yield losses of 50%, related to its larval foliage consumption activity^1^. The management of insect pests is one of the main challenges for agricultural researchers, and it’s difficultly increases with pest resistance and cross-resistance to chemical insecticides^2^. The negative impacts of pesticides on human health and environment have prompted more research on alternative control strategies for the integrated management of native and invasive pests^3–7^. To produce high-quality, pest-free crops without endangering the environment, researchers have focused on finding alternative, effective, and environmentally friendly control methods^8^. Botanicals are plant-derived materials that can be used as major components in integrated pest management (IPM) to control insect pests^9–12^ and reduce the use of synthetic insecticides. Thus, plant extracts are viable alternatives as they can regulate pest insect populations by affecting biological and behavioral parameters^13,14^.

Among the plant families that regulate insect pest populations, the previously tested behavioral and insecticidal properties of Magnoliaceae, Salicaceae, and Anacardiaceae were analyzed in the present study. Many studies have been performed on *Magnolia* species for their biphenolic phytochemicals magnolol and honokiol, which possess diverse pharmacological properties. Moreover, *Magnolia* extracts and their bioactive chemicals have been evaluated for potential insecticidal activity^15–17^. Different solvent extracts of *M. salicifolia* Maxim showed good to moderate larvicidal effects on fourth larvae of *Aedes aegypti*^18^.

Willows (*Salix* spp., family Salicaceae) are deciduous trees or shrubs well known for their medicinal effects. Many ancient civilizations used extracts of willow bark and willow leaf because of their analgesic, antipyretic, and anti-inflammatory properties^19,20^. Many studies have documented the presence of bioactive secondary compounds, such as polyphenols, terpenoids, and most importantly, salicylate compounds, in these plants^21–25^, which play a critical role not only as a part of their defense mechanisms and signaling molecules, but also as therapeutic agents (especially salicin)^26,27^. Plants synthesize salicylic acid (SA) through two pathways: the isochorismate pathway (IC) and the phenylalanine ammonia-lyase (PAL) pathway^28^. Willow is a well-known source of SA, which induces systemic resistance against several plant diseases^29^. Aqueous extracts of willow reduced *Fusarium* wilt in tomato seedlings by decreasing the level of lipid peroxidation^30^. The bark extract of the common willow has been approved by the EU pesticide regulations for agricultural applications as a basic substance with fungicidal properties^31,32^.

*Schinus terebinthifolia* (Anacardiaceae), commonly known as Brazilian pepper, has received particular attention owing to its nutritional, ornamental, and health-promoting properties, which can be attributed to a plethora of bioactive components, particularly phenols, tannins, flavonoids, saponins, alkaloids, and sterols^33–35^. *Schinus terebinthifolius* has insecticidal properties against *Stegomyia aegypti*^36^, *Anopheles gambiae, A. arabiensis*, and *Culex quinquefasciatus*^37^, *S. littoralis*^38^, and whitefly, *Bemisia tabaci*^39^. The essential oils of *S. terebinthifolius* fruits can also be used in the control of *S. littoralis* and *Phthorimaea operculella*, in association with IPM practices^40^.

Chitin synthesis inhibitors are insect growth regulators that affect insect chitin biosynthesis^41–43^. Novaluron is a benzoylphenylurea insecticide that interferes with developmental processes in immature insects, including abortive molting^44–46^. Novaluron ingested by adults can often be transferred transovarially to eggs, thereby reducing populations of economically important insect pests^45,47^. Moreover, it has low toxicity to mammals and several important natural enemies^48^.

Determining how digestive enzymes react to various inhibitors is a promising method to control phytophagous insects. There is limited published information on the inhibitors of *S. littoralis* digestive enzymes^49,50^.

The present study was conducted to evaluate the insecticidal activity of *M. grandiflora*,* S. terebinthifolius*, and *S. babylonica* extracts against different stages of *S. littoralis* (Boisd.). Furthermore, this study aimed to investigate the repellent and biological effects of these extracts on *S. littoralis* under laboratory or semi-field conditions; to determine the biochemical properties of extracts (e.g., in vitro inhibition of α-amylase and total protease activities); and to provide recommendations for using these plant-derived extracts in IPM programs to control this major pest.

## Materials and methods

### Insect rearing

This study has complied with relevant institutional, national, and international guidelines and legislation. This study does not contain any studies with human participants or animals performed by any of the authors. A laboratory strain of *S. littoralis* was reared on castor bean leaves, *Ricinus communis* L., under constant conditions of 27 ± 2 °C and 65 ± 5% relative humidity (RH), in the Insect Physiology Laboratory, Department of Applied Entomology and Zoology, Faculty of Agriculture, Alexandria University, Egypt. Moths were provided with *Nerium oleander* L. leaves for egg laying. Moreover, as the field strain, egg masses of *S. littoralis* were collected from cotton fields at El-Beheira Governorate, Egypt, and maintained in the laboratory under the aforementioned conditions.

### Test extracts

Extracts from *Magnolia grandiflora* leaves (Magnoliaceae), *Schinus terebinthifolius* wood (Anacardiaceae), and *Salix babylonica* leaves (Salicaceae) were used in this study. Novaluron (Equo® 10% EC; field rate, 60 mL/100 L water; Isagro Co., Italy) was used as a positive control for evaluating and comparing with the effectiveness of these extracts. All solvents and reagents used in experiments were analytical grade.

### Extraction procedure

Plant materials from the three tree species (*M. grandiflora* leaves, *S. terebinthifolius* wood, and *S. babylonica* leaves) were collected from Alexandria, Egypt. The collection of plants have been done and identified at the Department of Forestry and Wood Technology, Faculty of Agriculture, Alexandria University, Alexandria, Egypt. All plant materials were air-dried at room temperature for approximately 10 days and then ground to a powder using a small laboratory mill. Approximately 50 g of *Magnolia grandiflora* leaves was soaked in n-hexane (100 mL) in a conical flask for 3 days and then filtered through Whatman no. 1 filter paper. The solvent was evaporated using a rotary evaporator, and the n-hexane oily extract was concentrated.

For the extracts that were analyzed by HPLC, water and methanol extracts from *M. grandiflora* leaves and methanol extracts of *S. terebinthifolius* wood and *S. babylonica* leaves were used. Approximately 50 g of each ground material was soaked in 150 mL of solvent (water or methanol) for one week, then filtered through filter paper (Whatman no. 1), and concentrated by evaporating the solvent under reducing pressure with a rotary evaporator^51^. For the water extract of *M. grandiflora* leaves, a few drops of methanol were used to prevent any fungal growth. The content of the extracts was measured as a percentage per mass of the air-dried raw materials.

### HPLC analysis of extracts

For the phytochemical analysis, the phenolic compounds from extracts of *M. grandiflora* leaves, *S. terebinthifolius* wood*,* and *S. babylonica* leaves were identified by HPLC (Agilent 1100). The instrument was composed of binary LC pump, a UV/Vis detector, and C18 column (125 mm × 4.60 mm, 5-µm particle size)^52^.

### Toxicity tests

#### Ovicidal activity

Freshly deposited egg masses from the laboratory strain of *S. littoralis* were collected and counted using a hand lens (10 ×). Six concentrations of each extract and novaluron (0.5, 1, 2, 5, 10, and 20 mg/L) were prepared. Oleander leaves containing approximately 100 eggs were dipped for 20 s in each concentration of the test compounds separately. Another set of egg masses (100 eggs) on the oleander leaves was dipped in water to represent the control. Each concentration and control was replicated thrice. Treated and untreated egg masses were left to dry and maintained at 27 ± 2 °C, 65 ± 5% RH. After the maximum hatching time, the unhatched eggs in each treatment were counted using a binocular.

#### Larvicidal activity

The efficacy of the tested plant extracts and novaluron against the newly molted second and fourth larvae of *S. littoralis* was evaluated using a standard leaf-dip method. Six concentrations of each extract and insecticide (0.5, 1, 2, 5, 10, and 20 mg/L) were prepared. Castor bean leaves, which were almost equal in size, were dipped in the tested concentrations for 10 s and then left to dry. A set of castor leaves was dipped in distilled water only as the control. Each treatment was replicated thrice (20 larvae per treatment). The larvae were allowed to feed on treated leaves, and the mortality percentages were recorded 48 and 96 h post-treatment.

### Repellent effects of the tested materials

#### Feeding deterrence activity

The feeding deterrence effect of the *M. grandiflora* leaf water extract, *S. terebinthifolius* wood methanol extract, and *S. babylonica* leaf methanol extract and of novaluron insecticide against second and fourth larvae of *S. littoralis* was determined using the leaf disc method (no-choice test) 48 h post-treatment. Three concentrations of *M. grandiflora* extract (1, 5, and 10 mg/L), *S. terebinthifolius* and *S. babylonica* extracts (1, 2, and 5 mg/L), and novaluron (0.5, 1, and 2 mg/L) were used. These concentrations were chosen after the preliminary tests according to their effectiveness. The feeding deterrence index (FD %) was calculated using the following equation: FD % = [(C − T) / (C + T)] × 100; where C is the consumption of control discs and T is the consumption of treated discs^53^.

#### Anti-oviposition activity

A no-choice test was used to evaluate the effects of the tested extracts on the oviposition. The three above-mentioned concentrations of each plant extract and insecticide were used. Each pair (female and male) of newly emerged adults was placed in a glass jar with a ball of cotton dipped in a 10% sugar solution for feeding. Oleander leaves were treated with the test concentrations. The adults were left to feed, mate, and lay eggs on control and treated oleander leaves. Adults were removed two days after the beginning of egg laying, oleander leaves were carefully taken, and the number of eggs laid by each female was counted using a binocular. The anti-oviposition effect was calculated as follows^54^: Repellent index (RI %) = [(C − T) / (C + T)] × 100; where C is the number of eggs in the control, and T is the number of eggs in the treatment.

### Biological aspects

Bioactivity of the tested plant extracts and the insecticide was assessed under laboratory conditions (27 ± 2 °C, 65 ± 5% RH). The castor bean leaves were immersed separately in the above-mentioned concentrations of tested compounds or in distilled water for control, dried at room temperature, and transferred to petri dishes (12 cm in diameter). One hundred neonates (0–24 h) *S. littoralis* larvae were placed in each petri dish. Castor bean leaves were replaced with newly treated leaves every 24 h. To establish the pupation percentage and observe malformations, the larvae were continuously monitored until they reached the pupal stage. The pupae were sexed and transferred to 1-L glass containers (10 males and 10 females per container) to assess the percentage of adult emergence, mean number of eggs per female (fecundity), and hatchability.

### Biochemical assays

The in vitro inhibition of α-amylase and total protease activity were determined by incubating the prepared homogenate for 30 min at 37 °C with LC_50_ concentrations of the tested compounds prepared in distilled water containing the emulsifying agent (0.01% Triton-X 100). The control treatments were prepared by adding 0.01% Triton-X 100 without the tested compounds. Fourth larvae were then dissected, and midguts were excised, collected, and washed repeatedly with ice-cold saline solution (0.9% NaCl). The midguts were then homogenized in distilled water using a glass homogenizer surrounded with ice. The protein content was estimated by the method of Lowry et al.^55^ using bovine serum albumin as a standard protein to construct the standard curve.

### Alpha-amylase activity assay

The homogenate was centrifuged at 15,000 rpm for 15 min at 4 °C using IEC-CRU 5000 cooling centrifuge. The α-Amylase activity was estimated spectrophotometerically^56^. Fifty microliters of supernatant was added to 2.3 mM 2-chloro 4-nitrophenyl-α-Dmaltotrioside (CNPG3), 350 mM NaCl, 6 mM calcium acetate, 600 mM potassium thiocyanate, and 100 mM Good’s buffer (pH 6). An assay mixture without enzyme was used as a blank. The change in absorption at 405 nm was monitored using a Sequoia-Turner Model 340 spectrophotometer. The α-amylase activity was calculated as ΔOD_405_/mg protein/min.

### Total protease activity assay

The homogenate was centrifuged at 4000 rpm for 15 min at 4 °C in an IEC-CRU 5000 cooling centrifuge. The supernatant was used to estimate total proteolytic activity. Total protease activity was measured^57,58^ using azocasein as a substrate. The homogenate was incubated in a total volume 60 μL of assay buffer (100 mM Tris–HCl, pH 8) for 20 min at 37 °C before addition of 200 μL of 2% azocasein (w/v in assay buffer). After 180 min at 37 °C, the reaction was stopped by addition of 300 μL cold 10% trichloroacetic acid (TCA). The reaction mixture was centrifuged at 3000 rpm for 10 min in an IEC-CRU 5000 cooling centrifuge. Then, 10 μL NaOH (10 N) were added to the reaction mixture to neutralize excess acidity, and the absorbance was measured at 440 nm using a Sequoia-Turner Model 340 spectrophotometer. An assay mixture without homogenate was used as a blank. The total protease activity was calculated as ΔOD_440_/mg protein/min.

### Semi-field experiment

The residual toxicity of *M. grandiflora*, *S. terebinthifolius*, and *S. babylonica* extracts in comparison with novaluron against the field strain of fourth *S. littoralis* larvae was tested according to Raslan^59^ and El-Sheikh and Aamir^60^. Cotton seeds (*Gossypium barbadense* Linnaeus var. Giza 92) were sown in 50 plastic pots (30-cm in diameter) in the greenhouse of Cotton Pesticides Evaluation Department, Plant Protection Research Station, Alexandria, Egypt. Thirty days after emergence, the tested extracts and insecticide were applied as a foliar spray at a field rate (60 mL/100 L water) and a half field rate (30 mL/100 L water) using a hand-held sprayer with 1-L capacity until the leaves were saturated, and left to dry. The untreated plants were sprayed with tap water only. Cotton leaves from treated and untreated plants were randomly collected in perforated bags after 2 h of application and then 1, 2, 3, 4, and 7 days after the application, and transferred to the laboratory. Two cotton leaves from each sample were introduced to 20 newly molted fourth larvae of *S. littoralis* in Petri dish (12 cm in diameter) containing filter paper. Five replicates were performed for each treatment group. The Petri dishes were kept under laboratory conditions, at 27 ± 2 °C and 65 ± 5 RH %. The number of dead larvae was recorded, and mortality percentages were calculated 48 h after feeding.

### Statistical analysis

The LC_50_ and LC_95_ values for the toxicity tests were calculated using the Biostat ver. (2.1) software^61^ for probit analysis. Data were compared by one-way analysis of variance (ANOVA) followed by Tukey’s studentized test when significant differences were found at *P* < 0.05^62^.

## Results

### Chemical composition of the extracts

The extract contents from the studied plants were in *M. grandiflora* leaf water extract (8.12%), *M. grandiflora* leaf methanol extract (12.15%), *S. terebinthifolius* wood methanol extract (16.14%), and in *S. babylonica* leaf methanol extract (15.24%).

**Figure 1:**
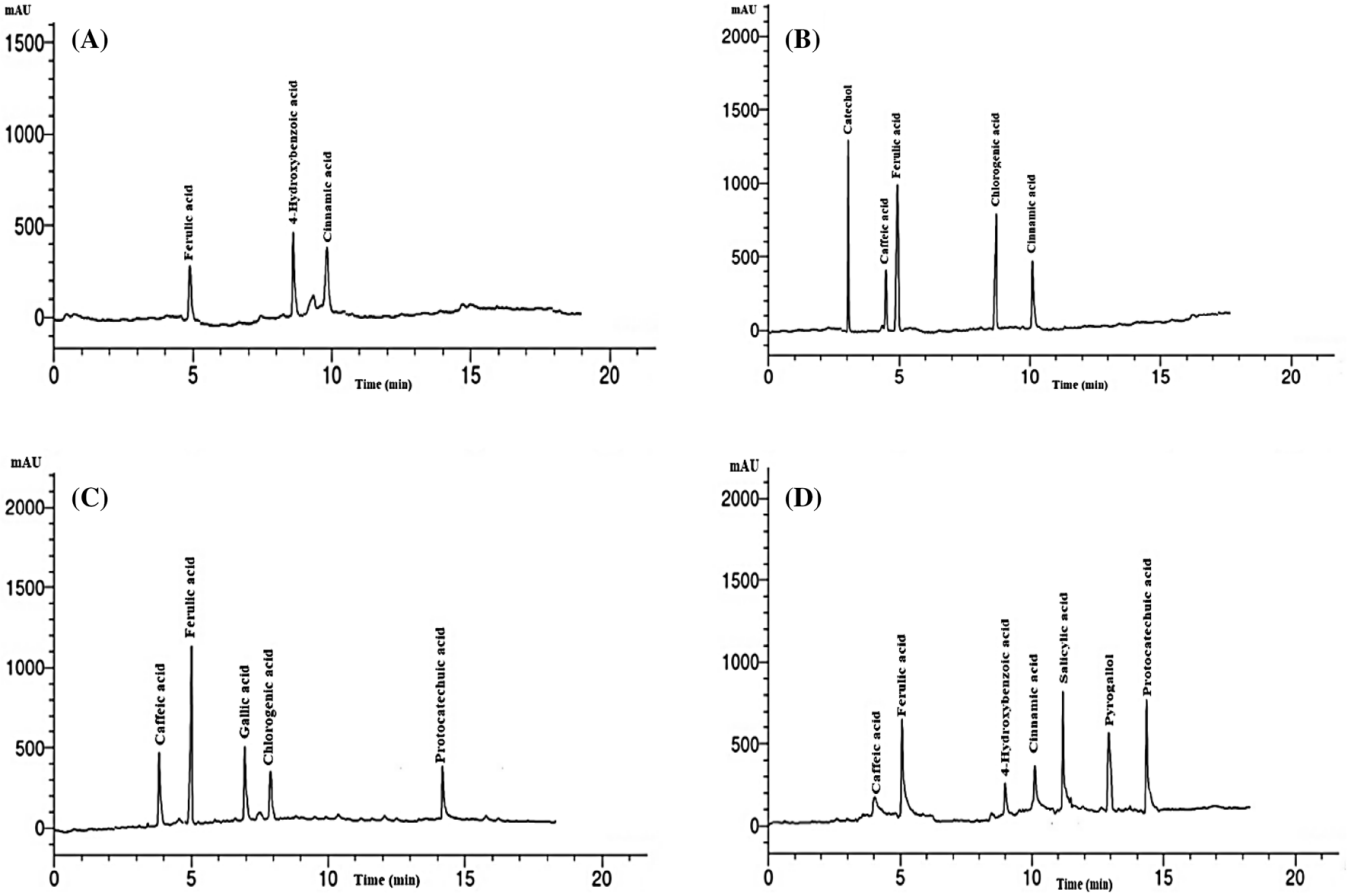
Chromatograms of High Performance Liquid Chromatography of the phenolic compounds identified in the extracts. (**A**) *M. grandiflora* leaf water extract; (**B**) *M. grandiflora* leaf methanol extract; (**C**) *S. terebinthifolius* wood methanol extract; (**D**) *S. babylonica* leaf methanol extract.

According to the HPLC analysis (Fig. 1 and Table 1), *M. grandiflora* leaf water extract (Fig. 1A) contained as main phenolic compounds 4-hydroxybenzoic acid (7.16 mg/mL), cinnamic acid (4.96 mg/mL), and ferulic acid (6.34 mg/mL), while the methanol extract (Fig. 1B) had catechol (13.05 mg/mL), ferulic acid (11.87 mg/mL), chlorogenic acid (10.33 mg/mL), and cinnamic acid (7.65 mg/mL). In the *S. terebinthifolius* wood extract (Fig. 1C), the phenolic compounds ferulic acid (14.81 mg/mL), caffeic acid (5.61 mg/mL), gallic acid (5.07 mg/mL), and chlorogenic acid (4.80 mg/mL) were abundant. In the methanol extract from *S. babylonica* leaves, (Fig. 1D) the phenolic compounds cinnamic acid (11.36 mg/mL), protocatechuic acid (10.33 mg/mL), ferulic acid (8.12 mg/mL), pyrogallol (8.05 mg/mL), and salicylic acid (6.44 mg/mL) were abundant.

**Table 1:** Phenolic compounds from extracts of *M. grandiflora* leaves, *S. babylonica* leaves and *S. terebinthifolius* wood by HPLC analysis.

Compound	Concentration (mg/mL)			
	*M. grandiflora* leaves		*S. terebinthifolius* wood	*S. babylonica* leaves
	Water	Methanol	Methanol	Methanol
Catechol	ND	13.05	ND	ND
Caffeic acid	ND	3.66	5.61	0.69
Ferulic acid	6.34	11.87	14.81	8.12
Gallic acid	ND	ND	5.07	ND
Chlorogenic acid	ND	10.33	4.80	ND
4-Hydroxybenzoic acid	7.16	ND	ND	1.23
Cinnamic acid	4.96	7.65	ND	11.36
Salicylic acid	ND	ND	ND	6.44
Pyrogallol	ND	ND	ND	8.05
Protocatechuic acid	ND	ND	3.69	10.33

*ND* not detected.

In the current study, the n-hexane extract of *M. grandiflora* leaves, which was analyzed by GC–MS had the following main compounds: palmitic acid, oleic acid, undecane, palmitoleic acid, (1-propyloctyl) benzene, (1-methyldecyl)-benzene, (1-ethylnonyl) benzene, (1-propylnonyl) benzene, stearic acid, (1-pentylhexyl) benzene, (1-ethyldecyl) benzene, and linoleic acid at 7.28%, 7.22%, 5.37%, 4.66%, 4.63%, 4.21%, 3.88%, 3.86%, 3.46%, 3.36%, 3.3%, and 3%, respectively^63^.

### Insecticidal effect of the tested extracts against *S. littoralis* stages

The insecticidal effects of the tested extracts were evaluated at different stages in *S. littoralis*. The toxic effects are shown as LC_50s_ values in Table 2. In general, the toxicity of the tested materials increased with time after treatment. Moreover, the instar larvae were the most sensitive stage to all tested materials.

**Table 2:** Toxicity of *Magnolia grandiflora* (water, methanol and n-hexane), *Schinus terebinthifolius*, *Salix babylonica* extracts and novaluron against different stages of *S. littoralis* after 48 and 96 h post-treatment.

Treatment	Stage	Time (h)	LC_50_^a^ (mg/L)	95% CL^b^	LC_95_^c^ (mg/L)	95% CL	Slope ± SE^d^	(χ^2^)^e^
*M. grandiflora* (Leaf water extract)	Egg	–	>10	–	–	–	–	–
	2nd larval instar	48	>10	–	–	–	–	–
		96	>10	–	–	–	–	–
	4th larval instar	48	>10	–	–	–	–	–
		96	>10	–	–	–	–	–
*M. grandiflora* (Leaf methanol extract)	Egg	–	>10	–	–	–	–	–
	2nd larval instar	48	>10	–	–	–	–	–
		96	>10	–	–	–	–	–
	4th larval instar	48	>10	–	–	–	–	–
		96	>10	–	–	–	–	–
*M. grandiflora* (leaf n-hexane extract)	Egg	–	>10	–	–	–	–	–
	2nd larval instar	48	>10	–	–	–	–	–
		96	>10	–	–	–	–	–
	4th larval instar	48	>10	–	–	–	–	–
		96	>10	–	–	–	–	–
*S. terebinthifolius* (Wood methanol extract)	Egg	–	0.94	0.68–1.20	17.06	10.72–34.82	1.31 ± 0.15	0.18
	2nd larval instar	48	1.65	1.29–2.06	17.53	11.12–35.09	1.60 ± 0.18	0.12
		96	0.89	0.72–1.10	21.31	13.32–46.68	1.73 ± 0.20	0.02
	4th larval instar	48	1.84	1.17–2.53	42.71	23.35–89.32	1.20 ± 0.18	0.16
		96	1.32	0.79–1.86	26.07	15.40–64.06	1.27 ± 0.19	0.03
*S. babylonica* (Leaf methanol extract)	Egg	–	1.05	0.82–1.31	12.11	7.99–22.38	1.55 ± 0.17	0.05
	2nd larval instar	48	1.64	1.32–2.08	17.53	11.12–35.09	1.64 ± 0.18	0.02
		96	1.02	0.74–1.33	13.72	8.52–29.36	1.46 ± 0.19	0.14
	4th larval instar	48	2.26	1.56–2.97	40.73	23.40–101.77	1.31 ± 0.17	0.09
		96	1.60	0.10–2.21	33.70	19.21–88.12	1.24 ± 0.18	0.03
Novaluron	Egg	–	0.62	0.49–0.78	6.77	4.52–12.24	1.58 ± 0.17	0.06
	2nd larval instar	48	1.23	0.95–1.56	30.33	17.23v74.63	1.62 ± 0.19	0.04
		96	0.55	0.41–0.70	14.58	9.06–30.98	1.55 ± 0.20	0.07
	4th larval instar	48	1.34	1.05–1.74	27.87	18.15–53.52	1.48 ± 0.19	0.08
		96	0.87	0.59–1.16	30.15	18.12–69.48	1.37 ± 0.19	0.22

^a^The concentration causing 50% mortality.

^b^Confidence limits.

^c^The concentration causing 95% mortality.

^d^Slope of the concentration-mortality regression line ± standard error.

^e^Chi square value.

### Ovicidal effect

The three extracts of *M. grandiflora* did not show any toxicity against *S. littoralis* eggs, but those of *S. terebinthifolius* and *S. babylonica* showed toxic effects. The highly toxic effect was shown by novaluron on eggs (LC_50_ = 0.62 mg/L). The *S. terebinthifolius* extract also exhibited high toxicity against *S. littoralis* eggs (LC_50_ = 0.94 mg/L) (Table 2).

### Larvicidal effect

*S. terebinthifolius* and *S. babylonica* showed toxic effects against second and fourth larvae after 48 and 96 h. Novaluron showed high toxicity against second larvae after 96 h (LC_50_ = 0.55 mg/L). Among the tested extracts, that of *S. terebinthifolius* had high toxic effect against second larvae after 96 h (LC_50_ = 0.89 mg/L). Compared with novaluron (positive control), the *S. terebinthifolius* wood methanol extract exhibited high toxicity against *S. littoralis* at different stages (Table 2).

As the three extracts of *M. grandiflora* did not show any toxicity against *S. littoralis* stages, the water extract was only used in subsequent behavioral, biological, and semi-field experiments to investigate whether it had any other effects on *S. littoralis*. Moreover, its use in IPM programs with different modes of action apart from toxicity was also tested.

### Repellent effects of the tested extracts

Phytophagous insects such as *S. littoralis* usually visit plants for either feeding or laying eggs. Identification of effective repellent or attractant agents for the early detection and suppression of *S. littoralis* populations is critical for managing this pest and reducing crop loss.

### Feeding deterrence activity

**Figure 2:**
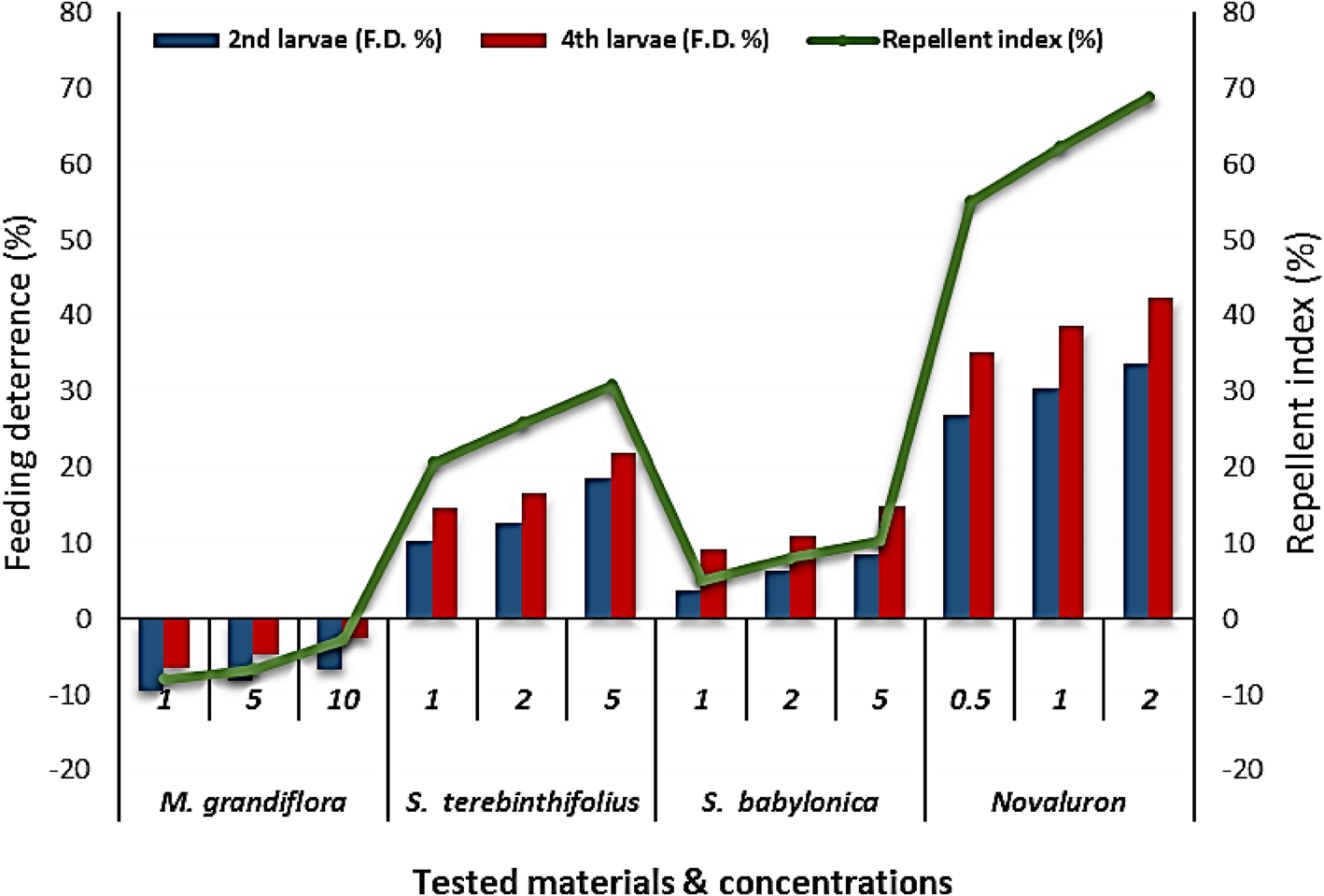
Anti-oviposition and antifeedant activities of *M. grandiflora*, *S. terebinthifolius*, *S. babylonica* extracts, and novaluron on egg laying and on second and fourth larvae of *Spodoptera littoralis* after 48 h of treatment (no-choice test).

The tested extracts were investigated for their feeding deterrent or attractant activity against second and fourth larvae of *S. littoralis*. All the tested materials showed feeding deterrence (FD %) values above the negative control, except for *M. grandiflora* extract, which was a feeding attractant for second and fourth larvae (Fig. 2). The fourth larvae were generally more affected by the tested compounds than the second larvae. Novaluron had a higher feeding repellent activity against *S. littoralis* larvae than all tested extracts. *S. terebinthifolius* wood methanol extract had a significantly higher feeding deterrence activity than the other extracts (FD % = 21.9% and 18.4% for fourth and second larvae, respectively, at 5 mg/mL). In contrast, *M. grandiflora* extract showed an attractant effect that decreased with increasing concentration (FD% =  − 9.6% and − 6.6% for fourth and second larvae, respectively, at 10 mg/L).

### Anti-oviposition activity

Insect oviposition is an important step in reproduction and in determining the size of a population. Therefore, deterrence of oviposition by a pest insect can decrease population size and assist in its management. The oviposition behavior of some phytophagous insects is altered by volatile products of host and non-host species. The deterrent or attractant activity of the tested materials against oviposition is shown as the repellent index (RI%) in Fig. 2. All tested compounds showed anti-oviposition activity, except for the *M. grandiflora* leaf water extract, which had RI% =  − 8%, -6.7% and -2.8% at 1, 5 and 10 mg/L, respectively.

### The impact of the tested compounds on some biological aspects

**Figure 3:**
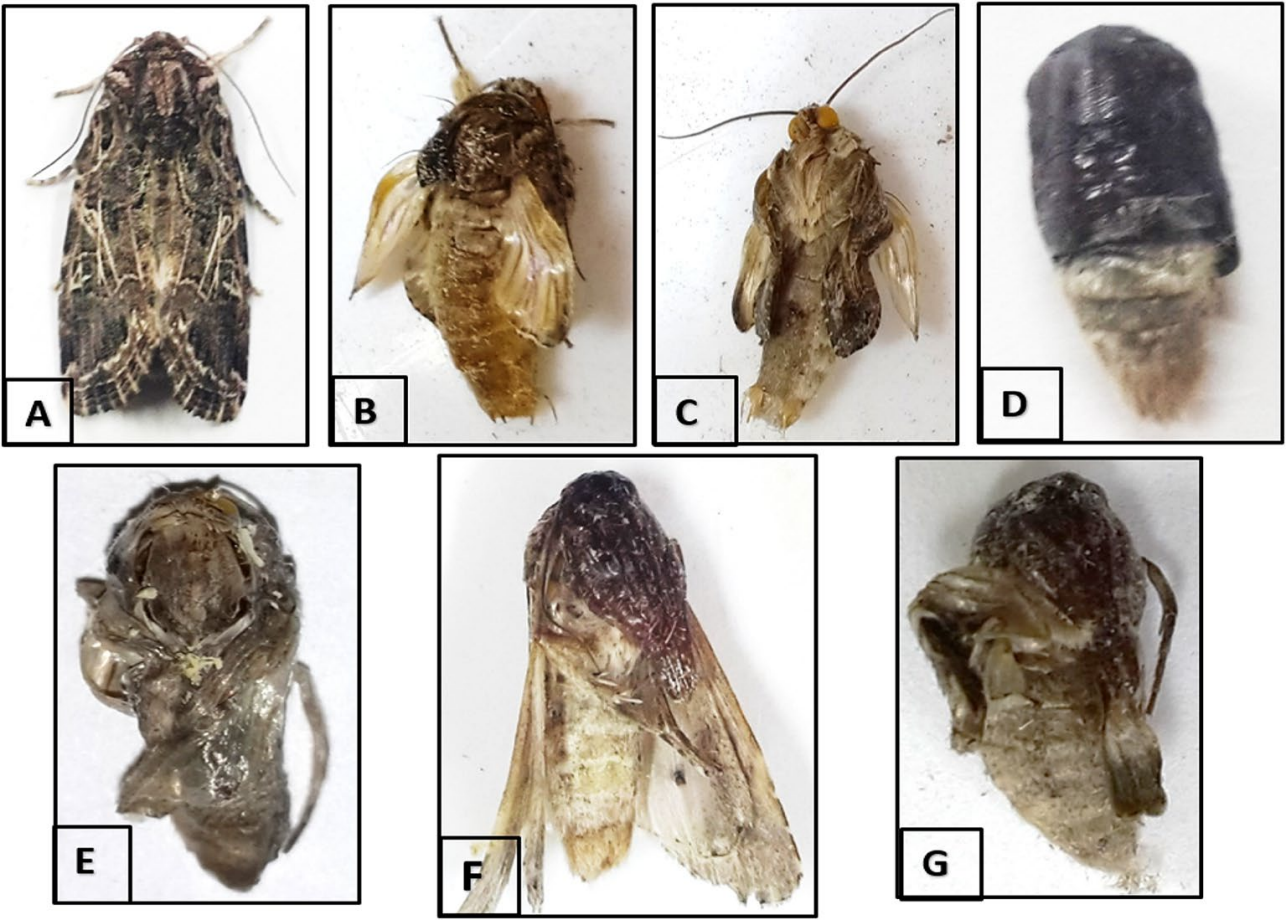
Malformations of *S. littoralis* adults affected by applications on larval stage. Control: normal adult (**A**). Adults resulting from larvae treated with 1 and 2 mg/L novaluron (**B** & **C**), with short, undeveloped legs and wings. Adults resulting from larvae treated with 2 and 5 mg/L *S. terebinthifolius* (**D**), which were unable to remove the old exoskeleton (exuvium) and (**E**) with abnormal and conjoined wings. Adults resulting from larvae treated with 2 and 5 mg/L *S. babylonica* (**F**), with head and mouthparts not completely molted, (**G**) with shrunken, folded, and undeveloped wings and preserved pupal head and mouthparts.

The results on pupation, adult emergence, fecundity (mean number of eggs/female), and hatchability (%) of the resulting eggs of the treated *S. littoralis* larvae are shown in Table 3. Adult growth disruption and abnormalities are shown in Fig. 3 (A–G). All tested materials affected the assessed biological aspects at all concentrations, except for the *M. grandiflora* extract, which affected larvae only at the highest concentration (10 mg/L). The biological effects of *S. terebinthifolius* were significantly reducing the percentages of pupation, adult emergence, hatchability, and fecundity (60.2%, 56.7%, 35.3%, and 105.4% eggs/female, respectively, at 5 mg/L).

**Table 3:** Effect of *M. grandiflora*, *S. terebinthifolius*, and *S. babylonica* extracts and of novaluron on pupation, adult emergence, egg production, and hatching percentages of *S. littoralis* after application on second larvae.

Treatment	Conc. (mg/L)	Pupation (%)	Adult emergence (%)	Number of eggs /female	Hatch (%)
Control	–	94.2 ± 1.2^a^	88.0 ± 1.3^a^	216.2 ± 5.2^a^	85.6 ± 1.8^a^
*M. grandiflora* (Leaf water extract)	1	93.8 ± 1.6^a^	87.3 ± 2.5^a^	210.3 ± 4.8^a^	84.3 ± 0.9^a^
	5	92.7 ± 2.1^a^	86.2 ± 1.8^a^	202.7 ± 5.6^ab^	83.5 ± 1.3^a^
	10	90.4 ± 1.7^b^	85.7 ± 3.4^ab^	196.5 ± 5.3^b^	80.6 ± 1.6^b^
*S. terebinthifolius* (Wood methanol extract)	1	66.0 ± 2.3^e^	63.3 ± 2.1^e^	128.6 ± 4.2^f^	48.0 ± 1.5^e^
	2	65.3 ± 1.9^e^	61.2 ± 2.7^ef^	119.2 ± 3.4^f^	44.7 ± 2.1^e^
	5	60.2 ± 3.2^f^	56.7 ± 3.8^f^	105.4 ± 3.1^g^	35.3 ± 0.8^f^
*S. babylonica* (Leaf methanol extract)	1	81.3 ± 3.3^c^	78.0 ± 3.2^c^	174.2 ± 2.5^cd^	66.2 ± 2.3^c^
	2	80.8 ± 2.8^c^	76.8 ± 1.4^c^	167.0 ± 4.3^d^	58.3 ± 1.2^d^
	5	75.0 ± 3.6^d^	69.3 ± 3.3^d^	154.6 ± 5.8^e^	54.6 ± 1.7^d^
Novaluron	0.5	56.3 ± 2.3^g^	48.4 ± 3.6^g^	54.8 ± 4.0^h^	24.3 ± 2.4^g^
	1	49.6 ± 1.5^h^	43.6 ± 4.2^h^	38.9 ± 3.4^i^	20.7 ± 2.3^g^
	2	47.2 ± 2.4^h^	41.3 ± 2.9^h^	26.4 ± 2.2^j^	14.3 ± 1.8^h^

Mean values ± standard error followed by the same letters in the same column are not significantly different at *P* < 0.05.

### Alpha-amylase and total protease activities of *S. littoralis* fourth instar larvae

**Figure 4:**
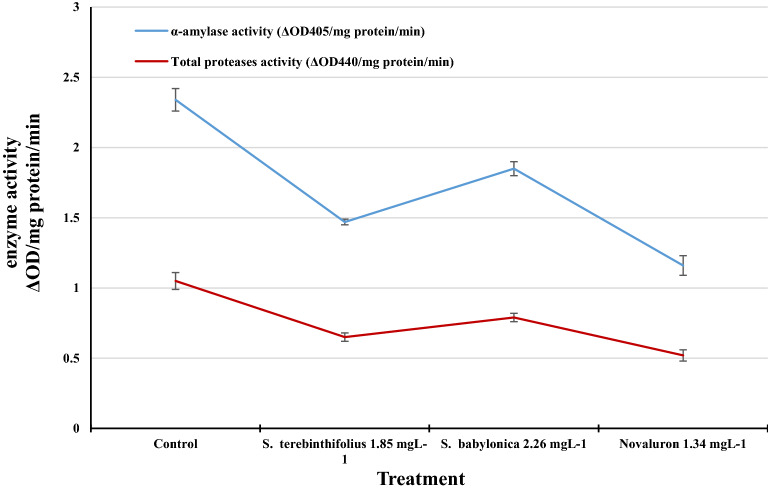
Effect of *S. terebinthifolius*, and *S. babylonica* extracts and of novaluron on the α-amylase and total proteases activities of the fourth *S. littoralis* larvae at LC_50_ value at 48 h post-treatment.

The significant inhibitory effects of LC_50_ *S. terebinthifolius*, *S. babylonica,* and novaluron on α-amylase and total proteases were determined in vitro (Fig. 4). Novaluron had the highest inhibition effect, with the activities of α-amylase and total proteases reduced to 1.16 and 0.52 ΔOD/mg protein/min, respectively, followed by the *S. terebinthifolius* extract, with activities reduced to 1.47 and 0.65 ΔOD/mg protein/min, respectively (compared with 2.34 and 1.05 ΔOD/mg protein/min in the control, respectively). The *S. terebinthifolius* extract reduced the activity of *S. littoralis* digestive enzymes.

### Residual toxicity of tested compounds against *S. littoralis* field strain

A semi-field experiment was conducted to evaluate the residual efficacy of *M. grandiflora*, *S. terebinthifolius*, *S. babylonica,* and novaluron against the fourth *S. littoralis* larvae from the field strain. The highest mortality percentages of *S. littoralis* larvae were 100%, 100%, 95%, and 70% after 2 h of application with novaluron, *S. terebinthifolius*, *S. babylonica*, and *M. grandiflora*, respectively. The mortality percentages decreased gradually over time to become 40.0%, 20.0%, 10.0%, and 0%, respectively, after 7 days of spraying at the field rate (60 mL/100 L water). While, the mortality percentages were 30%, 15%, 5%, and 0% at the half field rate (30 mL/100 L water) (Table 4).

**Table 4:** Residual toxicity of *M. grandiflora*,* S. terebinthifolius*, and *S. babylonica* extracts and of novaluron against the field strain of fourth *S. littoralis* larvae.

Treatment	Rate	Mortality percentages after the indicated periods from the application					
		2 h	1-day	2-days	3-days	4-days	7-days
*M. grandiflora* (Leaf water extract)	0.5 FR	60.0 ± 1.9^e^	40.0 ± 1.2^h^	25.0 ± 0.8^h^	15.0 ± 1.4^g^	5.0 ± 0.3^h^	0
	FR	70.0 ± 1.2^d^	55.0 ± 1.8^g^	35.0 ± 1.1^g^	25.0 ± 0.9^f^	10.0 ± 0.5^g^	0
*S. terebinthifolius* (Wood methanol extract)	0.5 FR	95.0 ± 1.3^b^	85.0 ± 1.7^d^	70.0 ± 1.3^d^	50.0 ± 1.7^c^	30.0 ± 0.6^d^	15.0 ± 0.3^d^
	FR	100.0 ± 0.0^a^	90.0 ± 1.4^c^	80.0 ± 2.1^c^	70.0 ± 1.2^b^	45.0 ± 1.3^c^	20.0 ± 0.8^c^
*S. babylonica* (Leaf methanol extract)	0.5 FR	85.0 ± 1.2^c^	75.0 ± 1.3^f^	45.0 ± 0.9^f^	30.0 ± 0.5^e^	15.0 ± 1.1^f^	5.0 ± 0.3^f^
	FR	95.0 ± 1.6^b^	80.0 ± 0.4^e^	60.0 ± 2.2^e^	35.0 ± 1.6^d^	25.0 ± 1.4^e^	10.0 ± 0.2^e^
Novaluron	0.5 FR	100.0 ± 0.0^a^	95.0 ± 0.8^b^	85.0 ± 1.3^b^	70.0 ± 0.9^b^	50.0 ± 1.6^b^	30.0 ± 0.5^b^
	FR	100.0 ± 0.0^a^	100.0 ± 0.0^a^	90.0 ± 0.6^a^	80.0 ± 1.2^a^	60.0 ± 0.8^a^	40.0 ± 0.9^a^

Mean values ± standard error followed by the same letters in the same column are not significantly different at *P* < 0.05. The mortality percentages were assessed after 48 h from the feeding. Field rate (FR) was applied at 60 ml/100 L water.

## Discussion

Insect pest management has always been and will remain a constant challenge for agricultural researchers and producers alike. There is an urgent need to replace pesticides with alternative control methods that are effective, inexpensive, and environmentally-friendly, therefore plant-derived products have received much attention in recent years due to drawbacks associated with unwise use of synthetic insecticides^8^.

Novaluron, which had already been shown to be highly toxic against *S. littoralis* eggs^64^, also showed a great ovicidal activity against *S. littoralis* eggs compared to the control. The strong insecticidal activity of *S. terebinthifoiuls* against *S. littoralis* has been previously reported^40^, as well as against *Anopheles gambiae*, *A. arabiensis*, and *Culex quinquefasciatus*^37^. *S. terebinthifolius* also showed high toxicity against two whitefly species, *Bemisia tabaci*, and *Trialeurodes ricini*^39^, *Aphis nerii* Boyer de Fonscolombe^65^ and *Plutella xylostella* (Lepidoptera: Plutellidae)^66^.

In contrast, *M. grandiflora* extract was herein found to have no toxic effect on *S. littoralis* stages*,* similar to the findings for *Magnolia citrata* essential oil, which had weak insecticidal activity against *S. littoralis* larvae compared with the positive control permethrin^38^. These results also agree with those of Vásquez-Morales and Flores-Estévez^67^, who found that the seed and sarcotesta extracts of *Magnolia schiedeana* (Magnoliaceae) only showed insecticidal activity against *Anastrepha ludens* adults, whereas the extracts of leaves, flowers, bark, and follicles showed no significant biological activity**.** Moreover, Ali et al.^68^ observed that the leaf, flower, and seed essential oils of *M. grandiflora* at the highest dose of 125 mg/L resulted in only 20%, 0%, and 50% mortality of *Aedes aegypti*, respectively.

Furthermore, *M. citrata* oil has been reported to exhibit weak toxicity against first instar larvae and adult female *A. aegypti*^69^. Methanol extract of *S. babylonica* leaves showed strong toxicity against *S. littoralis* stages, which is consistent with the results of Hasaballah et al.^70^, who showed that the toxic effects of methanol and ethanol extracts of *Salix safsaf* could compete with the synthetic insecticide deltamethrin as a natural insecticide in the control of the housefly *Musca domestica.* Added to the antifungal effect of salicin, the major compound of willow extract, other metabolites may increase the potency of willow extracts^71^.

The attractant effect of *Magnolia* was first reported by Pavela^38^, who reported the attractant activity of *M. citrata* essential oil on *S. littoralis*. The oil from *M. citrata* leaves has a moderately strong attractant effect on the sterile male medfly *Ceratitis capitata*^69^*.* In contrast, essential oils from five different parts of *M. grandiflora* showed biting deterrence against *Ae. aegypti*^68^. *Schinus terebinthifolius* produced a significantly high feeding deterrence activity in second and fourth instar larvae. The ethanolic extracts of *S. terebinthifolius* were effective antifeedants for third instar larvae of *Plutella xylostella* (Lepidoptera: Plutellidae)^72^. Treatment with *S. terebinthifolius* extract prolonged the larval phase, allowing a higher food intake before reaching the pupal stage; this probably resulted from the effect of one or several deterrent factors, resulting in nutritional imbalance and damage to the insect life cycle^73^. Willow oil generated maximum attraction (28.79%) in *Bemisia tabaci*, contrary to the repellent effect of willow oil on *S. littoralis*^74^.

Novaluron at 2 mg/L had the highest RI% (68.8%), followed by *S. terebinthifolius* extract at 5 mg/L (30.9%). Aly and Ali^64^ showed that novaluron had the highest oviposition deterrence value (23%) in *S. littoralis* females. Ethanolic extracts of *S. terebinthifolius* suppressed oviposition in *P. xylostella* adults^72^.

Novaluron also causes *S. littoralis* growth disruption and abnormalities, selectively targeting immature insect stages by inhibiting chitin formation and causing abnormal endocuticular deposition abortive molting^75^. Novaluron had the highest sterility value (68.9%) for *S. littoralis*^64^. The ethanolic extracts of *S. terebinthifolius* negatively affected all the evaluated biological parameters of *P. xylostella*, increasing the duration of the larval stage, which led to reduced pupal mass and oviposition period^76^. Treatment with *S. terebinthifolius* extract resulted in the lowest pupal mass and a greatest prolongation of the larval stage compared with other treatments^73^. Phytochemical studies on *S. terebinthifolius* have isolated tannins that inactivate digestive enzymes of insects, hampering their digestion, which in turn affects their growth and survival^73,77^. The *S. terebinthifolius* extract possibly reduced pupal survival by impairing their ability to feed as a result of larval sensitivity to the secondary compounds present in the plant extracts^78^.

It was previously explained that tannins act by inactivating the digestive enzymes in the leaves of *S. terebinthifolius*, generating a tannin-protein complex that is difficult to digest and affects the growth and survival of insects^73^. A reduction in food digestibility was observed in the tannin fractions of *S. terebinthifolius* by *Spodoptera frugiperda* larvae (Smith 1797) (Lepidoptera: Noctuidae)^79^.

Various studies have demonstrated that plant phenolic metabolites negatively affect insect feeding behavior, growth, development, and reproduction, and they may have lethal effects on specific insects^80,81^. Furthermore, α-amylase and protease activities of *S. littoralis* were decreased in the midgut after feeding on an artificial diet containing caffeic acid^82^. Ferulic acid, the most abundant phenolic compound from extracts of *M. grandiflora* leaves and *S. terebinthifolius* wood decreased adult emergence, delayed the developmental period and reduced the nutritional indices of *Spodoptera litura* (Fabricius) larvae^83^. The larval growth, survival, adult emergence, pupal weight, and different nutritional indices of *S. litura* (Fab.) were adversely affected by the various concentrations of purified phenolic compounds like chlorogenic acid^84^. The effect of some phenolic acids (chlorogenic, caffeic, and ferulic) on the growth, development and midgut enzyme activities of *S. litura* larvae was studied through diet incorporation assay and can be utilized in insect control programs^85^. The two cinnamic acid derivatives were found to show higher levels of insecticidal, larvicidal and larval growth inhibition activities against *Tribolium castaneum*^86^. The lethal effect of chlorogenic acid on *Mythimna separata* (Walker) (Lepidoptera: Noctuidae) and that a sublethal concentration harmed larval growth and development^87^.

## Conclusion

The extract of *S. terebinthifolius* can be used to efficiently manage *S. littoralis*, as they had various modes of action, such as toxicity, repellence, growth regulation, reduction of fecundity, and inhibition of digestive enzymes activity. Therefore, they can be considered suitable alternatives and can be incorporated into IPM systems for *S. littoralis*.

## Data Availability

All data generated or analyzed during this study are included in this published article.
